# Iron: Protector or Risk Factor for Cardiovascular Disease? Still Controversial

**DOI:** 10.3390/nu5072384

**Published:** 2013-07-01

**Authors:** Carlos Muñoz-Bravo, Mario Gutiérrez-Bedmar, Jorge Gómez-Aracena, Antonio García-Rodríguez, Joaquín Fernández-Crehuet Navajas

**Affiliations:** Department of Preventive Medicine and Public Health, University of Málaga, Boulevard Louis Pasteur, 32, Málaga 29071, Spain; E-Mails: carlosmb@uma.es (C.M.-B.); gomezaracena@uma.es (J.G.-A.); antoniogr@uma.es (A.G.-R.); crehuet@uma.es (J.F.-C.N.)

**Keywords:** iron, body iron stores, cardiovascular diseases, myocardial infarction

## Abstract

Iron is the second most abundant metal in the Earth’s crust. Despite being present in trace amounts, it is an essential trace element for the human body, although it can also be toxic due to oxidative stress generation by the Fenton reaction, causing organic biomolecule oxidation. This process is the basis of numerous pathologies, including cardiovascular diseases (CVD). The relationship between iron and cardiovascular disease was proposed in 1981 by Jerome Sullivan. Since then, numerous epidemiological studies have been conducted to test this hypothesis. The aim of this review is to present the main findings of the chief epidemiological studies published during the last 32 years, since Sullivan formulated his iron hypothesis, suggesting that this element might act as a risk factor for cardiovascular disease. We have analyzed 55 studies, of which 27 supported the iron hypothesis, 20 found no evidence to support it and eight were contrary to the iron hypothesis. Our results suggest that there is not a high level of evidence which supports the hypothesis that the iron may be associated with CVD. Despite the large number of studies published to date, the role of iron in cardiovascular disease still generates a fair amount of debate, due to a marked disparity in results.

## 1. Introduction

**Figure 1:**
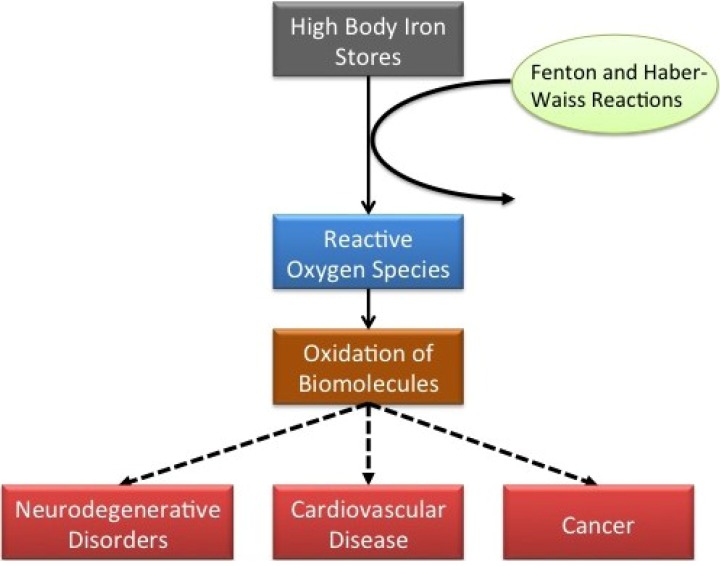
Oxidative stress due to high body iron stores.

Iron is the second most abundant metal in the Earth’s crust. Despite being present in trace amounts, it is an essential trace element to the human body involved in cellular processes and a key component of various enzymes. However, an excess of iron can be toxic because it has the ability to accept and donate electrons by exchanging between ferrous and ferric forms. This exchanging may generate reactive oxygen species through Fenton and Haber-Weiss reactions, causing oxidative stress and organic biomolecule oxidation. This process is at the basis of pathologies like neurodegenerative disorders, cardiovascular diseases or cancer (Figure 1).

More than 30 years have passed since Sullivan published his iron hypothesis [1], arguing that the greater number of cases of cardiovascular disease (CVD) in men and postmenopausal women, *versus* premenopausal women, was due to these women having lower iron deposits as a result of menstrual bleeding. The hypothesis was supported by the following observations: (a) myocardial failure in iron storage diseases; (b) accumulation of stored iron with age in men; and (c) accumulation of stored iron after menopause to levels found in men. Despite the studies that have been conducted to date, Sullivan’s hypothesis is still the subject of debate. The aim of this review is to present the main conclusions of the most important epidemiological studies to date, classified in two groups according to the association with CVD, depending on the type of study and year of publication, and sorted from oldest to most recent.

## 2. Studies Finding an Association between Iron and CVD

### 2.1. Cross-Sectional Studies

Kiechl *et al.* (1994) [2] analyzed the relation between sonographically assessed carotid atherosclerosis and body iron stores in a population sample of 847 men and women. The study population was composed of an age- and sex-stratified random sample of 1000 men and women aged 40–79, with a participation rate of 93.6%. In a logistic regression analysis adjusting for age, sex, and all major vascular risk markers, ferritin was one of the strongest indicators of carotid artery disease in both sexes (odds ratio (OR) = 1.54 per 100 μg/L; *p* < 0.001). The predictive significance of ferritin was found to be synergistic with that of hypercholesterolemia.

Haidari *et al.* (2001) [3] studied 400 Iranian patients (218 males, 182 females) referred for diagnostic coronary angiography, and recorded risk factors for coronary artery disease (CAD), lipids, C-reactive protein (CRP), and ferritin concentrations. A multiple logistic regression analysis, after adjusting for the established coronary risk factors, showed ferritin as an independent discriminating risk factor for CAD (*p* < 0.01). Men in the highest quartile of ferritin had an OR = 1.62; 95% Confidence Interval (CI): 1.12–2.42, compared with men in the lowest quartile of ferritin. It is, however, remarkable that they did not find any association between ferritin and CAD among the female patients. Discussing this lack of association in women, the authors indicated that a higher ferritin concentration is required to contribute to the progression of atherosclerotic lesions in women.

Wolff *et al.* (2004) [4] assessed intima-media thickness and plaque prevalence in the carotid arteries by high-resolution ultrasound among 2443 participants (1200 women aged 45–79) in a population-based study in Northeast Germany. In the multivariate analysis, serum ferritin levels were not independently associated with carotid intima-media thickness among women or men. In contrast, the relationship between serum ferritin levels and carotid plaque prevalence was significant among men (OR = 1.33; 95% CI: 1.08–1.44), yet not among women. However, the researchers also found a dose-response relation between serum ferritin levels and carotid plaque prevalence, in which odds ratios (ORs) for carotid plaque prevalence became greater with increasing serum ferritin levels, similar for women and men.

In a cross-sectional study, Zheng *et al.* (2005) [5] analyzed the effects of blood donation frequency on body iron stores and physiological and biochemical biomarkers of vascular function associated with atherosclerosis progression in 40 high-frequency voluntary blood donors (≥8 donations in the past two years) and 42 low-frequency blood donors (1–2 donations in the past two years), aged 50–75 and randomly selected from the American Red Cross of Connecticut’s blood donor records. Serum ferritin was significantly decreased in high-frequency blood donors when compared with low-frequency blood donors (median values 17 ng/mL *versus* 52 ng/mL; *p* < 0.001), although hematocrit did not differ between groups. Seventeen of the 40 high-frequency blood donors had evidence of severely reduced iron stores (serum ferritin < 12 ng/mL) compared with two of the 42 low-frequency blood donors (44% *versus* 5%; *p* < 0.001). The total iron binding capacity (TIBC) was significantly increased in the high-frequency blood donors when compared with the low-frequency blood donors (mean ± standard error of the mean (SEM) = 363 ± 10 µg/dL *versus* 325 ± 7 µg/dL; *p* = 0.003). Serum levels of 3-nitrotyrosine, a marker of oxidative stress, were significantly decreased in high-frequency donors when compared with low-frequency donors (35 *versus* 43 nmol/L; *p* < 0.02). Flow-mediated dilation was significantly increased in high-frequency blood donors when compared with that of low-frequency blood donors (mean ± SEM = 5.5 ± 2.6% *versus* 3.8 ± 1.6%, *p* < 0.001). In the multivariate analysis, high-frequency blood donation remained significantly associated with increased flow-mediated dilation when adjusting for established traditional and non-traditional cardiovascular risk factors. Based on these results, the researchers concluded that high-frequency blood donation was associated with evidence of reduction of body iron stores, improved vascular function, and reduced oxidative stress in voluntary blood donors.

Menke *et al.* (2009) [6] studied the association of serum ferritin and transferrin saturation (TS) with the prevalence of peripheral arterial disease (PAD), assessed through the ankle-brachial blood pressure index, in 1631 men and 1031 postmenopausal women. After stratifying the analysis by cholesterol levels, the authors found a modest association of ferritin and TS with PAD, particularly among those with high cholesterol levels (≥200 mg/dL). For the male subjects, the multivariable adjusted ORs were 1.30 (95% CI: 0.99–1.72) for serum ferritin and 2.59 (95% CI: 0.99–6.78) for TS. For the female subjects, adjusted ORs were 1.20 (95% CI: 0.95–1.51) for serum ferritin and 2.07 (95% CI: 1.01–4.22) for TS.

Rajapurkar *et al.* (2012) [7] studied the association between the so-called catalytic iron, *i.e.*, chemical forms of iron that can participate in redox cycling, and CVD in a cross-sectional study of 496 participants. The odds of existing CVD for subjects in the upper third of catalytic iron were 10 times that of subjects with lower catalytic iron in unadjusted analyses. In the final model that adjusted for age, the Framingham Risk Score, estimated glomerular filtration rate, hypertension status, high-density lipoprotein cholesterol (HDL-cholesterol), and systolic blood pressure, the OR was decreased to 3.8, but the association remained significant (OR = 3.8, 95% CI: 1.4–10.1). Therefore, the authors reported a direct association between serum catalytic iron and prevalent CVD, after controlling for multiple known atherosclerosis risk factors.

Syrovatka *et al.* (2012) [8] evaluated the relationship between body iron stores (the plasma-circulating transferrin receptor concentration to plasma ferritin concentration ratio (TfR/F) and ferritin) and asymptomatic atherosclerosis (by carotid intima-media thickness measured by ultrasound) in a study that included 72 healthy men. Both the TfR/F ratio and ferritin showed significant association with common carotid intima-media thickness (*r* = −0.310, *p* = 0.008 *versus r* = 0.295, *p* = 0.012). The multivariate analysis confirmed that the correlation of TfR/F with common carotid intima-media thickness is independent of traditional atherosclerosis risk factors (β = −0.334, *p* = 0.037). The findings of these authors showed a clear association of body iron stores, expressed by the TfR/F ratio, with asymptomatic carotid atherosclerosis in a cohort of healthy men.

In 2012, Sung *et al.* [9] analyzed an occupational cohort of 12,033 South Korean men, who underwent a cardiac computed tomography estimation of coronary artery calcium score (CACS) and measurements of multiple cardiovascular risk factors, whether any relationship between ferritin and CACS > 0 (as a marker of atherosclerosis) was independent of potential confounders, such as iron binding capacity (transferrin), low-grade inflammation, and cardiovascular risk factors. The main results were: (a) the median interquartile range serum ferritin concentration was 196.8 (136.3–291.9) ng/mL in people with a CACS > 0, compared with 182.2 (128.1–253.6) ng/mL in people with a CACS = 0 (*p* < 0.001); (b) in the highest ferritin quartile, 14.7% of the subjects (442/3008) had a CACS > 0 compared with 9.7% of the subjects (292/3010) in the lowest quartile; (c) in the logistic regression analysis, the OR for the presence of CACS > 0 in the highest quartile of ferritin concentration, *versus* the lowest quartile, was 1.66 (95% CI: 1.39–1.98). Finally, these researchers concluded that ferritin concentrations were associated with the presence of CAC, regardless of traditional cardiovascular risk factors, including the Framingham Risk Score, transferrin, preexisting vascular disease, diabetes mellitus, metabolic syndrome factors, and low-grade inflammation.

Recently, Leiva *et al.*(2013) [10] investigated the association between oxidative stress markers and iron nutrition status in subjects (ages ranging from 45 to 65 years old) with metabolic syndrome (MetS). Of the 155 subjects randomly selected, 85 were classified with MetS and 70 without the syndrome (no MetS). Compared to the lowest quartile of ferritin, there was a threefold higher risk of developing MetS in the highest quartile of ferritin in analyses adjusted for sex, BMI (body mass index), and hs-CRP (highly sensitive C-reactive protein) (OR = 3.36; 95% CI: 1.14–4.20). Both men and women with MetS showed higher concentrations of serum ferritin (μg/L) than no MetS subjects (72.4 (46.8–111.5) *versus* 55.4 (35.6–96.3), *p* < 0.001; 53.9 (34.1–84.8) *versus* 27.4 (12.6–59.5), *p* < 0.001, respectively) and total body iron (mg/kg) (mean ± standard deviation (SD) = 8.3 ± 2.8 *versus* 7.0 ± 2.1, *p* < 0.04; 6.3 ± 2.3 *versus* 4.1 ± 2.8, *p* < 0.001, respectively). Besides, men and women with MetS showed lower levels of the transferrin receptor (μg/mL) than no MetS subjects (mean ± SD = 4.8 ± 2.1 *versus* 5.8 ± 1.0, *p* < 0.03; 6.0 ± 1.8 *versus* 7.2 ± 2.9, *p* < 0.004, respectively). On the other hand, both men and women in the MetS group showed significantly higher levels of oxidative stress parameters (heme oxygenase activity, oxidized low density lipoprotein (LDL), thiobarbituric acid reactive substances) than the no MetS group. On the basis of these results, the researchers concluded that an elevated plasma concentration of oxidative stress parameters, such as oxidized LDL, thiobarbituric acid reactive substances, and iron storage, were associated with metabolic syndrome.

### 2.2. Case-Control Studies

Regnström *et al.* (1994) [11] performed a case-control study in Swedish men to analyze the connection between iron metabolic parameters (serum ferritin, serum iron, and serum TIBC) and MI. Ninety-four consecutive men with a first MI before the age of 45 were studied 4–6 months after the infarction, along with 100 age-matched, population-based male controls. Serum ferritin (µg/L) was higher in patients than in controls (mean ± SD = 138.8 ± 90.0 and 137.5 ± 90.4 respectively), although not significantly. Serum TIBC (µmol/L) was significantly higher in patients than in controls (mean ± SD = 61.05 ± 7.82 and 56.78 ± 5.69, *p* < 0.001, respectively). Therefore, serum iron (µmol/L) was significantly lower in patients than in controls (mean ± SD = 20.42 ± 6.00 and 23.07 ± 6.30, *p* < 0.01, respectively). These data suggest that low stored iron levels are a risk factor for premature coronary atherosclerosis and MI in young Swedish men.

Tuomainen *et al.* (1998) [12] investigated the association of the TfR/F ratio with the risk of acute myocardial infarction (AMI) in a prospective nested case-control study in men from Eastern Finland. Transferrin receptor assays were carried out for 99 men who had an AMI during a median follow-up of 6.4 years and 98 male controls. Both the cases and the controls were nested from a cohort of 1931 men who had no clinical coronary heart disease (CHD) at the baseline study. The controls were matched for age, examination year, and residence. The mean serum TfR/F ratio was 28.6% (95% CI: 2.2%–54.8%) lower among the cases than the controls (*p* = 0.035 for difference in *t* test). In a multivariate logistic regression model adjusting for other strongest risk factors for AMI (like smoking, HDL, and LDL cholesterol) and indicators of inflammation and alcohol intake, a low TfR/F ratio was significantly associated with an increased risk of AMI (*p* =0.040). Based on these results, the authors suggested that men with high body iron stores (low TfR/F ratio) had a twofold to threefold increased risk of the first AMI.

In a nested case-control study of 60 patients who had suffered their first myocardial infarction (MI) and 112 age- and sex-matched control subjects, Klipstein-Grobusch *et al.* (1999) [13] found that the risk of MI associated with the highest ferritin tertile was most evident in current or former smokers (OR = 1.68; 95% CI: 1.17–2.47; *p*-trend = 0.008) and in subjects with hypercholesterolemia (OR = 1.43; 95% CI: 0.99–2.11; *p*-trend = 0.056) or diabetes (OR = 2.41; 95% CI: 1.12–7.67; *p*-trend = 0.027). The authors concluded that this association between ferritin and MI might be increased in patients with other risk factors.

Kervienen *et al.* (2004) [14] studied the association between serum iron and CHD in a nested case-control study with 215 cases and 215 matched controls over 8.5 years. The coronary risk (OR) in subjects with low iron (<17 µmol/L) was 2.1 (95% CI: 1.1–3.8). The subjects with low iron, high-sensitivity C-reactive protein (hs-CRP), and a high total leukocyte count were at an increased risk (OR = 9.8; 95% CI: 3.9–24.4). A mix of a high herpes simplex virus-1 antibody level and high hs-CRP in subjects with low iron increased the risk (OR = 13.1; 95% CI: 2.9–60.1). According to the results, the authors suggested an association between low serum iron level and coronary risk. The association is not independent, but is related to the fact that chronic infections and inflammation are accompanied by low serum iron.

In a prospective nested case-referent study, Ekblom *et al.* (2011) [15] investigated the connection between iron status (measured as plasma iron, TIBC, TS, and ferritin) and the risk of suffering a first MI. The authors found an inverse risk association for MI in the highest quartiles of iron (OR = 0.68; 95% CI: 0.48–0.96) and TS (OR = 0.62; 95% CI: 0.42–0.89) in men. This association, however, was lost after adjusting for CRP. Nevertheless, after adjusting for age, body mass index, systolic blood pressure, smoking, hs-CRP, Apo B/Apo A1 ratio, and diabetes, the authors found an inverse association again for MI in the highest quartile of iron (OR = 0.62; 95% CI: 0.39–0.97) and TS (OR = 0.56; 95% CI: 0.34–0.89), compared with the lowest quartile of iron. So, the researchers concluded that iron levels in the upper normal range seemed to be associated with a lower risk for first MI.

Other authors have also researched into the possibility that body iron stores may be related to factors associated with CVD. Meroño *et al.* (2011) [16] analyzed the lipid and lipoprotein metabolism and novel markers of CVD in 20 male patients with iron overload (IO), *versus* 20 sex- and age-matched healthy controls, as well as their relationship with ferritin concentration and insulin resistance. IO was diagnosed based on: TS > 45%, ferritin concentration > 500 μg L^−1^ and homozygosity for HFE gene C282Y or H63D mutations, or increased iron liver stores assessed by semi-quantitative grading in liver biopsies. The main findings of this study included the presence of the so-called atherogenic dyslipemia in most patients with IO, apart from an increased oxidized LDL concentration and higher CETP (cholesteryl ester transfer protein) and lipoprotein-associated phospholipase A2 (Lp-PLA2) activities, in comparison with age- and sex-matched controls. Moreover, IO patients presented a reduction in the activity of the PON 1 (antioxidant enzyme paraoxonase 1). In addition, multiple regression analyses identified homeostasis model assessment-insulin resistance (HOMA-IR) as an independent predictor of CETP activity, as well as ferritin concentration of Lp-PLA2 activity. Based on these results, the authors concluded that IO patients presented not only insulin resistance but also metabolic alterations related to elevated iron stores and associated with a high risk of CVD.

In a hospital based case-control study, Holay *et al.* (2012) [17] studied the relationship between serum ferritin and AMI. A total of 75 cases and an equal number of controls were studied. The median serum ferritin values were significantly higher in cases (220 µg/L), as compared to controls (155 µg/L) (*p* < 0.001). In a multivariate analysis, high serum ferritin (>200 µg/L) acted as an independent risk factor associated with MI (OR = 5.72; 95% CI: 2.16–15.17). Therefore, these researchers found a strong independent relationship between high serum ferritin and AMI.

In 2012, Kang *et al.* [18] studied the effect of TIBC on CAD risk in a hospital-based case-control study with 258 CAD cases and 282 healthy controls. The TIBC concentration was significantly lower in cases (mean ± SD = 85.05 ± 36.60 μmol/L) than in controls (mean ± SD = 118.59 ± 61.35 μmol/L) (*p* < 0.001). The multivariate-adjusted OR for the highest quartile, compared with the lowest quartile, was 0.08 (95% CI: 0.04–0.18). The authors concluded that TIBC was negatively associated with CAD risk.

### 2.3. Retrospective Cohort Study

In a retrospective cohort study of men older than 39 and women older than 50, Meyers *et al.* (2002) [19] analyzed the effect of blood donation on cardiovascular events in a frequent donor group (1508 adults who donated more than 1 unit of whole blood each year between 1988 and 1990) and in a casual donor group (1508 age- and sex-matched adults who donated only a single unit over the same 3-year period). Events occurred in 6.3% of frequent and 10.5% of casual donors. After adjusting for group differences, the OR was 0.60, 95% CI: 0.43–0.83. Among participants who had not donated blood before 1988, the adjusted OR was 1.88, 95% CI: 0.62–5.69, compared to those who had previously denoted blood (adjusted OR = 0.49; 95% CI: 0.35–0.68). The beneficial effect of frequent donation was greater in women than in men (adjusted OR = 0.25; 95% CI: 0.09–073 *versus* OR = 0.67; 95% CI: 0.47–0.94, respectively). Based on these results, the investigators suggested that frequent and long-term whole blood donation might be associated with a lower risk of cardiovascular events.

### 2.4. Prospective Studies

In 1992, Salonen *et al.* [20] developed a prospective study in a randomly selected group of Finnish men (*n* = 1931), aged 42, 48, 54, or 60, who had no symptomatic CHD at entry. After performing a Cox proportional hazards model adjusting for a number of variables like age, examination year, cigarette pack-years, ischemic ECG in exercise test, maximal oxygen uptake, systolic blood pressure, and blood glucose, among others, men with serum ferritin greater than or equal to 200 µg/L had a 2.2-fold (95% CI 1.2–4.0) risk factor-adjusted risk of AMI, compared with men with a lower serum ferritin. Dietary iron intake also had a significant association with MI in a Cox model with the same covariates.

Morrison *et al.* (1994) [21] assessed the relation of serum iron, dietary iron, and use of iron supplements to the risk of fatal AMI among both men and women in a cohort study with 9920 participants. Both men and women in the highest serum iron category (≥175 µg/dL) experienced an increased risk for fatal AMI (males: rate ratio = 2.18; 95% CI: 1.01–4.74; females: rate ratio = 5.53; 95% CI: 1.69–18.12). Nevertheless the authors found no important association between either dietary iron consumption or taking iron supplements and risk of fatal AMI.

In a large prospective survey (4-year follow-up), Ascherio *et al.* (1994) [22] analyzed the association between iron intake and the risk of coronary disease in 44,933 US men (with no previous history of CVD) aged 40–75. Using a food frequency questionnaire at baseline, 844 incident cases of coronary disease were documented. After adjusting for established risk factors, no association was found between total iron intake and dietary intake of heme iron with CHD risk. However, the incidence of fatal coronary disease or non-fatal MI was higher among men in the top quintile of heme iron intake, compared with men in the lowest quintile (Relative Risk (RR) = 1.42; 95% CI: 1.02–1.98). Thus they found an increased risk of MI among men with a higher intake of heme iron.

Sempos *et al.* (1994) [23] examined the relation between iron status, measured by the serum TS, and CHD risk and overall mortality. A total of 4518 men and women were studied. The authors used a multivariate Cox proportional hazards model. Base-line data were collected from 1971 to 1974, with follow-up through 1987. TS was used as a measure of the amount of circulating iron. Higher TS levels were not associated with an increased risk of CHD or MI. A significant inverse association with TS was found for overall mortality and for mortality from cardiovascular causes in white men and women.

Liao *et al.* (1994) [24] found that serum iron was inversely associated with MI risk in women, but not in men. TIBC and TS were not related to MI in either sex, while serum iron and TS were inversely associated with CHD in both sexes. Dietary iron intake based on 24-h recall was not associated with the two disease endpoints.

Magnusson *et al.* (1994) [25] investigated the association between iron and MI in a randomly selected group (*n* = 2036) of Finnish men and women aged 25–74 since 1983 in a follow up of 8.5 years. In the study, 81 subjects experienced AMI (63 men and 18 women). Using the Cox proportional hazards model for estimating the contribution of independent variables to MI risk, TIBC was found to be a strong independent negative risk factor in men (RR = 0.94; 95% CI: 0.91–0.98). Whereas ferritin and MI were not associated (RR = 0.99; 95% CI: 0.99–1.00).

Kiechl *et al.* (1997) [26] investigated the potential association between serum ferritin concentrations and the 5-year progression of carotid atherosclerosis as assessed by ultrasonographic follow-up evaluations. The study population comprised a random sample of 826 men and women aged 40–79. The authors found that serum ferritin emerged as one of the strongest risk indicators of the overall progression of carotid atherosclerosis when the analysis was adjusted for baseline vascular status (atherosclerosis score), age, sex, risk attributes, and alcohol consumption (OR = 1.50/SD unit; *p* < 0.001).

Corti *et al.* (1997) [27] examined the association between serum iron and CAD, CVD, and all-cause mortality in a large cohort of 3936 subjects aged ≥71, surviving at least one year after baseline. The median follow-up time was 4.4 years. The authors found a gradual decrease in the relative risks (RRs) of CAD, CVD, and all-cause mortality with increasing serum iron levels (all tests for trend, *p* < 0.05). Furthermore, there was consistent evidence of an increasing risk of mortality at lower serum iron levels. Lower serum iron levels were associated with an increased risk of CAD, CVD, and all-cause mortality. Men in the lowest quartile of iron were five times more likely to die of CAD than men in the upper quartile (RR = 0.22; 95% CI: 0.11–0.48), while women in the lowest quartile of iron had a risk of CAD death that was twice that of women in the upper quartile (RR = 0.48; 95% CI: 0.27–0.87). Results of similar strength and magnitude were observed for CVD mortality in both sexes.

Salonen *et al.* (1998) [28] tested the hypothesis that the donation of blood might theoretically reduce the risk by lowering body iron stores. A cohort of 2862 men aged 42–60 were monitored for an average of almost nine years. In a Cox proportional hazards model adjusting for age, examination years, and all other predictive coronary disease risk factors, blood donors had a 88% reduced risk (relative hazard (RH) = 0.12; 95% CI: 0.02–0.86) of AMI, compared with non-blood donors.

In a prospective study (from 1993 to 1997), van der A *et al.* (2005) [29] researched the relationship between iron intake and CHD risk in a cohort comprising 17,357 women aged 49–70. For iron intake, a validated food frequency questionnaire (FFQ) estimated the usual frequency of consumption of 79 main food items over the preceding 12 months. They found that, after adjusting for cardiovascular and nutritional risk factors, menstrual periods, and antioxidant intake, a high dietary heme iron intake was associated with a 65% increase in CHD risk (hazard ratio (HR) = 1.65; 95% CI: 1.07–2.53).

Van der A *et al.*(2006) [30] tested the relation of non-transferrin bound iron (NTBI), serum iron, TS, and serum ferritin with CHD and AMI in 11,471 postmenopausal women aged 49–70. During a median follow-up of 4.3 years, 185 first fatal and non-fatal CHD events occurred, of which 66 were AMIs. A weighted Cox proportional hazards model was used to estimate hazard ratios (HRs) for tertiles of iron variables in relation to CHD and AMI. The adjusted HRs obtained for the second and third tertiles of serum variables, compared with the lowest tertiles, showed no association between the aforementioned variables and CHD events. Nevertheless, the authors did observe a statistically significant inverse association of NTBI and serum iron with AMI risk (HR = 0.47; 95% CI: 0.31–0.72; HR = 0.49; 95% CI: 0.25–0.94, respectively). Albeit not statistically significant, TS and serum ferritin were also inversely related to AMI risk.

In a paper published in 2012, Zhang *et al.* [31] researched the association between dietary iron (assessed at baseline by a validated FFQ) and mortality from CVD in a large prospective study of 58,615 healthy Japanese (23,083 men and 35,532 women), aged 40–79, who had no history of strokes, CHD, or cancer at baseline. Dietary intake of total iron was positively associated with mortality from total and ischemic strokes and total CVD in men. The multivariable HR for the highest *versus* the lowest quintile of total iron intake was 1.43 (95% CI: 1.02–2.00; *p*-trend = 0.009) for total stroke and 1.27 (95% CI: 1.01–1.58; *p*-trend = 0.023) for total CVD in men. In women, dietary total iron intake was not associated with any endpoints.

In 2012, Kim *et al.* [32] reexamined the association of serum ferritin and TS (%) with all-cause, cancer, and cardiovascular mortality in men aged ≥50 years and postmenopausal women from 1988–1994. Serum ferritin was not associated with all-cause, cancer, or cardiovascular mortality in either men or postmenopausal women. However all-cause, cancer, and cardiovascular mortality were inversely associated with TS in men and for all-cause and cardiovascular mortality in postmenopausal women. These associations were statistically significant.

### 2.5. Clinical Trials

In a sub-study of a clinical trial designed to test the hypothesis that reducing body iron stores through phlebotomy will influence clinical outcomes in patients with symptomatic PAD [33], DePalma *et al.* (2010) [34] studied correlations between ferritin, inflammatory biomarkers and mortality in a cohort of 100 cancer-free patients with PAD. The authors found that ferritin levels were positively correlated with interleukin-6 levels (*r* = 0.185, *p* = 0.002) and high-sensitivity C-reactive protein levels (*r* = 0.118, *p* = 0.040). Furthermore, ferritin levels were significantly higher in patients who died than survivors (mean ± SD = 132.5 ± 116.8 and 83.6 ± 57.3, *p* = 0.05, respectively). The authors concluded that iron-induced oxidative stress may relate to inflammatory responses in patients with PAD.

Several studies have been derived from data of the above mentioned clinical trial [33], in which a total of 1277 participants with PAD were randomly assigned to iron reduction group (phlebotomy, *n* = 636) or control group (*n* = 641). Randomization was stratified according to five variables. Zacharski *et al.* (2011) [35] analyzed the effects of age and ferritin (both were randomization variables) on primary (all-cause mortality) and secondary (death, non fatal infarction, and stroke) outcomes. Iron reduction improved outcomes in youngest age quartile patients (primary outcome HR = 0.44; 95% CI: 0.21–0.92; secondary outcome HR = 0.34; 95% CI: 0.19–0.61). For the entire cohort, improved outcomes occurred with mean follow-up ferritin levels below the median of the cohort means (primary outcome HR = 1.48; 95% CI: 1.14–1.92; secondary outcome HR = 1.22; 95% CI: 0.99–1.50). The authors concluded that lower iron burden predicted improved outcomes overall and was enhanced by phlebotomy. Recently, DePalma *et al.* (2013) [36] have published a study comparing the outcomes between smokers and non-smokers (another randomization variable). Iron reduction resulted in significant improvement in the primary and secondary outcomes (HR = 0.66; 95% CI: 0.45–0.97 and HR = 0.64; 95% CI: 0.46–0.88 respectively) compared with controls in smokers but not in non-smokers. Based on the results, authors concluded that phlebotomy-related outcomes favored smokers over non-smokers. A very interesting relationship between the use of statins and ferritin levels was found recently by Zacharski *et al.* (2013). [37] Using the same data [33], the authors compared effects of ferritin levels *versus* high-density lipoprotein to low-density lipoprotein (HDL/LDL) ratios (both were randomization variables) on clinical outcomes in participants receiving and not receiving statins. The authors found that statins increased HDL/LDL ratios and reduced ferritin levels by noninteracting mechanisms. Furthermore, improved clinical outcomes were associated with lower ferritin levels but not with improved lipid status.

Houschyar *et al.* (2012) [38] studied the effects of phlebotomy and the controlled reduction of body iron in patients with MetS in a randomized, controlled, single-blind clinical trial. Of the 64 patients with metabolic syndrome, 33 were randomly assigned to iron reduction by phlebotomy and 31 to a control group. Primary outcomes were change in systolic blood pressure and insulin sensitivity as measured by the homeostatic model assessment (HOMA) index after six weeks. Secondary outcomes included glycosylated hemoglobin A1c (HbA1c), plasma glucose, blood lipids, and heart rate. Systolic blood pressure decreased from (mean ± SD) 148.5 ± 12.3 mmHg to 130.5 ± 11.8 mmHg in the phlebotomy group, and from (mean ± SD) 144.7 ± 14.4 mmHg to 143.8 ± 11.9 mmHg in the control group (*p* < 0.001). No significant effect on the HOMA index was detected. With regard to the secondary outcomes, blood glucose, HbA1c, LDL/HDL ratio, and heart rate were significantly decreased by phlebotomy (*p* < 0.001). Changes in blood pressure and the HOMA index correlated with ferritin reduction (*r* = 0.39; *p* = 0.03). Therefore, the authors concluded that in patients with MetS, phlebotomy with a moderate reduction of body iron stores lowered blood pressure and resulted in improvements of markers of cardiovascular risk and glycemic control.

## 3. Studies Which Did not Find Any Association between Iron and CVD

### 3.1. Cross-Sectional Study

Solymoss *et al.* (1994) [39] studied whether serum ferritin concentrations are related to angiographically determined CAD or to a past history of MI in 225 men and 74 women, most of who were of French-Canadian origin. There were no significant differences in ferritin levels (µg/L) between men with ≥50% diameter stenosis (*n* = 195) and those with intact or minimally affected arteries (*n* = 31) (mean ± SD = 175 ± 121 *versus* 189 ± 189, *p* = 0.883). The same occurred in women with ≥50% diameter stenosis (*n* = 48) and those with intact or minimally affected arteries (*n* = 26) (mean ± SD = 91 ± 77 *versus* 109 ± 72, *p* = 0.192). There was no correlation between the quartiles of serum ferritin and the severity of CAD (Pearson’s χ^2^-test = 3.157, *p* = 0.788). There were no differences in ferritin levels in men with (*n* = 95) or without (*n* = 71) a history of MI (mean ± SD = 189 ± 188 *versus* 175 ± 121, *p* = 0.883). The same occurred in women with (*n* = 25) or without (*n* = 43) a history of MI (mean ± SD = 94 ± 82 *versus* 103 ± 64, *p* = 0.330). Based on these results, the authors concluded that serum ferritin levels were not related to the presence or severity of angiographically determined CAD or to a history of MI.

### 3.2. Case-Control Study

Mänttäri *et al.* (1994) [40] studied the relationship between ferritin and CVD in a nested case-control study in dyslipidaemic middle-aged men. Of the 140 subjects with cardiac endpoints (non-fatal MI or cardiac death), 136 were matched with controls for geographical area and drug treatment (gemfibrozil-placebo). In the logistic regression analyses, the adjusted OR in the highest serum ferritin tertile compared to the lowest was 0.78; 95% CI: 0.39–1.54; *p*-trend = 0.5. So, the authors concluded that serum ferritin was not a CHD risk factor, at least in dyslipidaemic middle-aged men.

Moore *et al.* (1995) [41] probed the association between serum ferritin and carotid intima-media thickening in a matched case-control study. For a serum ferritin concentration greater than 143 µg/L (the interquartile range), the OR for cases with carotid intima-media thickening *versus* controls was 1.12 (95% CI: 0.97–1.30). However, there was no association after adjusting for major cardiovascular risk factors. The authors concluded that these results did not support the hypothesis that increased iron stores increase the risk of atherosclerotic CVD.

From 1992 to 1994, Eichner *et al.* (1998) [42] studied the relationship between CAD (measured through coronary angiography) and iron binding proteins levels (ferritin and transferrin) in a consecutive series of white male (*n* = 457) and female (*n* = 114) cardiac patients in two Oklahoma hospitals. No association was found.

Endbergs *et al.* (1998) [43] studied the relationship between the extent of CAD and parameters of oxidation in 275 patients (208 men aged (mean ± SD) 55.1 ± 9.6 years and 67 women aged (mean ± SD) 54.6 ± 10.0 years) who underwent coronary angiography or percutaneous transluminal coronary angioplasty for the first time. They measured iron, ferritin, transferrin, copper, caeruloplasmin, and lipid. Coronary scores were assessed by the use of three scores (vessel score, stenosis score and extent score). The authors found an association between CAD and total cholesterol and LDL-cholesterol (*p* < 0.001) in women, LDL-cholesterol (*p* < 0.05) in men, and patient age showed a significant correlation with all three scores: although none of the parameters of oxidative metabolism (iron, transferrin, ferritin, copper, caeruloplasmin) correlated significantly with any of the three scores.

Tang *et al.* (2003) [44] compared five microelements in human serum, hair and fingernails in a diseased group and in a healthy control group. The following samples were taken from the aged hypertension and coronary heart disease group: serum (*n* = 57: 32 males and 25 females), hair (*n* = 46: 24 males and 22 females), and fingernails (*n* = 53: 28 males and 25 females). The mean age was (mean ± SD) 67.5 ± 10.4 years. The following samples were taken from the healthy control group: serum (*n* = 52: 28 males and 24 females), hair (*n* = 46: 23 males and 20 females), and fingernails samples (*n* = 49: 25 males and 24 females). The mean age was (mean ± SD) 63.6 ± 8.7 years. Analysis of the elements was performed using absorption spectrometry. Results for iron were significantly higher in the serum of the patients (mean ± SD = 1.37 ± 0.59 *versus* 1.02 ± 0.45; *p* < 0.01), but significantly lower in hair (mean ± SD = 45.9 ± 9.3 *versus* 53.3 ± 10.7; *p* < 0.01) and fingernails (mean ± SD = 42.2 ± 18.5 *versus* 64.6 ± 28.3; *p* < 0.001).

In 2004, Braun *et al.* [45] analyzed the association between serum ferritin or soluble transferrin receptor (sTfR) and CAD in 892 subjects who underwent coronary angiography. Of these, 664 were classified as cases and 228 as controls. The ferritin concentrations and sTfR (median, 25th–75th percentiles) were higher in cases than controls (140.1, 74.8–248.3 ng/ml *versus* 120.1, 74.9–218.0 ng/mL, *p* = 0.11; 2.6, 2.1–3.2 mg/L *versus* 2.4, 2.1–3.0 mg/L, *p* = 0.13 respectively); however this values were not statistically significant. In a multiple logistic regression model, ferritin and sTfR didn’t correlate independently with the presence of CAD. The investigators therefore concluded that there was no association between ferritin or sTfR and CAD.

Shi *et al.* (2011) [46] explored in a Chinese Han population the association between body iron stores and CHD, using a case-control study that involved 1334 CHD patients and 1334 age- and sex-frequency matched controls. The plasma ferritin levels in CHD cases (197.9 µg/L (2.7–932.9 µg/L)) were higher than those in controls (179.9 µg/L (21.1–878.2 µg/L); *p* = 0.028). The ORs across the tertiles of plasma ferritin levels were 1.0 (reference), 0.93 (95% CI: 0.76–1.13), and 1.23 (95% CI: 1.02–1.48); *p*-trend = 0.028. However, adjustment for the traditional risk factors attenuated the associations to null: 1.0 (reference), 0.90 (95% CI: 0.71–1.13), and 1.14 (95% CI: 0.91–1.43); *p*-trend = 0.22. Based on these results, the researchers concluded that there was no positive association of body iron store and CHD risk in Han Chinese.

### 3.3. Prospective Studies

Baer *et al.* (1994) [47] studied the association between body iron stores and AMI in 46,932 members of a prepaid health plan who were ≥30 years old and had received a standard health check between 1969 and 1971. Blood collected during this examination was analyzed for serum iron and TIBC. TS was categorized as low, normal or elevated. Hospital stays for AMI were identified through December 1991. The mean follow-up time was 14.1 years. During follow-up, 969 men and 871 women were treated in the hospital for AMI. The RRs obtained from proportional hazards regression adjusting for age, race, education, smoking, alcohol consumption, family history of AMI, personal history of diabetes, serum glucose, systolic blood pressure, cholesterol level, and body mass index for TS (%) were 1.8; 95% CI: 0.9–3.7 for low TS (≤10) and 1.5; 95% CI: 0.9–2.7 for high TS (≥62) and, therefore, not statistically significant.

In a prospective population study with a mean mortality follow-up time of 14 years, Reunanen *et al.* (1995) [48] studied whether increased body iron stores (assessed as TIBC and TS) and dietary iron intake (assessed by the dietary history during the previous year) were associated with an increased risk of CHD mortality. The study included 6086 men and 6102 women aged 45–64 at the baseline examination, without known heart disease. No relationship between TIBC and coronary mortality was observed in men; in women, an inverse although not significant association was found. TS was inversely but not significantly associated with coronary mortality in men; in women, the relationship was U-formed with a higher mortality at both the lower and higher ends of the distribution. Adjustment for other risk factors did not alter the results. No association was found with dietary iron intake and coronary mortality. Based on these results, the researchers concluded that high iron stores and increased iron intake were not associated with an increased risk of coronary disease.

In a 17-year follow-up study of 260 non-institutionalized elderly people aged 64–87, van Asperen *et al.* (1995) [49] explored the relationship between increased iron stores and the risk of ischaemic heart disease mortality. Haemoglobin levels, TS and TIBC were evaluated. The results found no association between increased iron stores and CHD mortality in either men or women.

Marniemi *et al.* (1998) [50] studied a random sample of 344 individuals aged 65 or older. Calcium, magnesium, copper, caeruloplasmin, zinc, selenium, iron, ferritin, transferrin, alpha-tocopherol, and other elements and vitamins were analyzed in blood specimens. After a 13 year follow-up, the relations between the compounds measured and relative mortality risks were analyzed by the Cox proportional hazards model, adjusting for other known risk factors. After adjustment for age, sex, body mass index, CHD, hypertension, diabetes, serum cholesterol, HDL-cholesterol and triglycerides, no association between either iron or ferritin and vascular disease risk was found.

Manfroi *et al.* (1999) [51] studied in 307 patients (60.9% male) the relationship between serum ferritin levels and the presence of coronary atherosclerosis. Of the patients, 196 (63.8%) had angiographic defined CAD and 111 (36.2%) no significant CAD. The comparison of ferritin quartiles between groups showed a significant association between serum ferritin and angiographic significant CAD (*p* = 0.015). However, when the predictors of angiographic significant CAD were used in a logistic regression model, the association between ferritin levels and significant CAD was no longer statistically significant (*p* = 0.27). So, serum ferritin levels did not reflect the coronary angiographic manifestation of this process. Finally, the researchers found no independent relationship between serum ferritin and angiographic CAD.

The relationship between serum ferritin and CVD, CHD and MI was also studied by Sempos *et al.* (2000) [52] in a prospective cohort study performed in the US. Data were collected from 1976 to 1980. The analytic sample (*n* = 1604) included 128 black men, 658 white men, 100 black women, and 718 white women, aged 45–74 at baseline, who, based on self-reported data, were free of CHD at baseline. Black men with a serum ferritin level of <0.50 µg/L had a significantly higher adjusted risk of death from all causes (RR = 3.1; 95% CI: 1.5–6.5). The authors did not find any other association between serum ferritin and the aforementioned endpoints. However, they described an apparent but non-significant U-shaped association between serum ferritin and all-causes mortality in black men and between serum ferritin and CVD death in white women.

Ascherio *et al.* (2001) [53] examined the association between blood donation and risk of CHD in a large prospective investigation of US men. In this study, 38,244 men free of diagnosed CVD were followed for four years. During this time, 328 nonfatal MI and 131 coronary deaths were documented. The age-adjusted RR of MI for men in the highest category of blood donations (≥30), compared with never-donors, was 1.2 (95% CI: 0.8–1.8), and this RR was not materially changed after adjusting for several coronary risk factors (1.3; 95% CI: 0.8–1.9).

Knuiman *et al.* (2003) [54] studied the association between serum ferritin levels and CHD and stroke events in a long-term Western Australian prospective study. The study took place from 1981–1998 and consisted of 1612 men and women aged 40–89, free of CVD at baseline. After adjusting for age and other CVD risk factors using proportional hazards regression models, the results did not show any evidence of a relationship between ferritin and CHD (HR = 0.95; 95% CI: 0.60–1.53) or between ferritin and strokes (HR = 1.43; 95% CI: 0.77–2.63).

During a 10-year period, Friedrich *et al.* (2009) [55] followed 2874 subjects with serum ferritin levels between 15 and 300 µ/L. During the follow-up period, 310 subjects (201 men; 109 women) suffered from CVD. Using multivariable Cox proportional hazards regression models (which included traditional CVD risk factors), no association was found. Analyses in women with serum ferritin levels categorized into three groups (low, <40; middle, 40–80; high, >80 µg/L) confirmed the assumption of a U-shaped relationship. Women with low or high serum ferritin levels tended to have a higher risk of CVD (low serum ferritin: HR = 1.92; 95% CI: 1.03–3.59; high serum ferritin: HR = 1.33; 95% CI: 0.85–2.08) and ischaemic heart disease (low serum ferritin: HR = 1.90; 95% CI: 0.68–5.28; high serum ferritin: HR = 1.55; 95% CI: 0.75–3.21), compared with women in the intermediate ferritin group. However, only one of these estimates was statistically significant. Sullivan (2001) [56] discussed this, indicating two possible ways of ferritin acting as a risk factor of CVD. Small amounts of iron may lead to maximal promotion of CVD and if high serum ferritin is a marker of persistent iron repletion, high levels might act as a risk factor. This concept is in agreement with these results of a U-shaped association in women.

Similarly, another study concluded that within the spectrum of normal iron metabolism, ferritin and TS were not associated with the risk of mortality among people who were not taking iron supplements and did not have a baseline history of CVD or cancer (Menke *et al.* (2012)) [57].

### 3.4. Clinical Trial

Zacharski *et al.* (2007) [33] tested the hypothesis that reducing body iron stores through phlebotomy would influence clinical outcomes in a cohort of patients with symptomatic PAD. The study was a multicenter, randomized, controlled, single-blinded trial conducted within the Department of Veterans Affairs Cooperative Studies Program from 1999 to 2005. Patients were assigned to control or iron-reduction groups through stratified randomization according to the participating hospital, age (≤60 and >60 years), ferritin level at entry (calculated based on the rolling mean of prior entrants), diagnosis of diabetes mellitus, smoking status, and ratio of HDL-cholesterol level to that of LDL-cholesterol (also calculated based on the rolling mean of prior entrants). Randomization was performed using the adaptive allocation method balanced on the marginal total of each factor. Of the 1277 patients entered from 24 participating medical centers, 641 were randomly assigned to the control group and 636 to the iron-reduction group. The primary endpoint was all-cause mortality; the secondary endpoint was death, plus non-fatal MI and stroke incidence. No statistically significant difference between treatment groups was observed for either the primary endpoint (HR = 0.85; 95% CI: 0.67–1.08) or the secondary endpoint (HR = 0.88; 95% CI: 0.72–1.07). Therefore, no association was found between phlebotomy and the aforementioned endpoints.

### 3.5. Meta-Analysis

In a meta-analysis of 12 prospective studies (involving a total of 7800 cases), Danesh and Appleby (1999) [58] evaluated the epidemiological association between iron status and CHD. Several of the studies reported on more than one marker of iron status. Five prospective studies of CHD and serum ferritin were identified. Overall, the comparison of individuals with serum ferritin measurements ≥200 *versus* those <200 μg/L at baseline yielded a combined risk ratio for CHD of 1.03 (95% CI: 0.83–1.29). Five other prospective studies involved TS and CHD. On the whole, the comparison of individuals with TS values in the top third with those in the bottom third at baseline yielded a combined risk ratio for CHD of 0.92 (95% CI: 0.74–1.14). Four studies reported on TIBC. Altogether, the comparison of individuals with levels of TIBC in the top third *versus* those in the bottom third at baseline yielded a combined risk ratio for CHD of 0.98 (95% CI: 0.66–1.46). Three studies reported on serum iron. Overall, the comparison of individuals with serum iron values in the top third *versus* those in the bottom third at baseline yielded a combined risk ratio for CHD of 0.83 (95% CI: 0.67–1.03). Finally, three other studies reported on total dietary iron intake. On the whole, the comparison of individuals with a total daily iron intake in the top third *versus* those in the bottom third at baseline yielded a combined risk ratio for CHD of 0.84 (95% CI: 0.66–1.06). Based on these results, the authors concluded that the published prospective studies did not provide good evidence to support the existence of strong epidemiological associations between iron status and CHD.

## 4. Conclusions

This review presents the main findings of the chief epidemiological studies published during the last 32 years, since Sullivan formulated his iron hypothesis, suggesting that this element might act as a risk factor for CVD. Table 1, Table 2 show the studies which support and do not support the iron hypothesis respectively.

**Table 1:** Studies which support the iron hypothesis.

STUDY DESING	AUTHOR	YEAR	REFERENCE
Cross-sectional	Kiechl *et al.*	1994	[2]
	Haidari *et al.*	2001	[3]
	Wolff *et al.*	2004	[4]
	Zheng *et al.*	2005	[5]
	Menke *et al.*	2009	[6]
	Rajapurkar *et al.*	2012	[7]
	Syrovatka *et al.*	2012	[8]
	Sung *et al.*	2012	[9]
	Leiva *et al.*	2013	[10]
Case-control	Toumainen *et al.*	1998	[12]
	Klipstein-Gobrusch *et al.*	1999	[13]
	Meroño *et al.*	2011	[16]
	Holay *et al.*	2012	[17]
	Kang *et al.*	2012	[18]
Retrospective Cohort	Meyers *et al.*	2002	[19]
Prospective Cohort	Salonen *et al.*	1992	[20]
	Morrison *et al.*	1994	[21]
	Ascherio *et al.*	1994	[22]
	Magnusson *et al.*	1994	[25]
	Kiechl *et al.*	1997	[26]
	Salonen *et al.*	1998	[28]
	Van der A *et al.*	2005	[29]
	Zhang *et al.*	2012	[31]
Clinical Trial	DePalma *et al.*	2010	[34]
	Zacharski *et al.*	2011	[35]
	Houschyar *et al.*	2012	[38]
	DePalma *et al.*	2013	[36]

**Table 2:** Studies which do not support the iron hypothesis.

STUDY DESING	AUTHOR	YEAR	IRON AND CVD RELATIONSHIP	REFERENCE
Cross-sectional	Solymoss *et al.*	1994	None	[39]
Case-control	Regnström *et al.*	1994	Inverse	[11]
	Mänttäri *et al.*	1994	None	[40]
	Moore *et al.*	1995	None	[41]
	Eichner *et al.*	1998	None	[42]
	Enbergs *et al.*	1998	None	[43]
	Tang *et al.*	2003	None	[44]
	Braun *et al.*	2004	None	[45]
	Kervinen *et al.*	2004	Inverse	[14]
	Ekblom *et al.*	2011	Inverse	[15]
	Shi *et al.*	2011	None	[46]
Prospective Cohort	Baer *et al.*	1994	None	[47]
	Sempos *et al.*	1994	Inverse	[23]
	Liao *et al.*	1994	Inverse	[24]
	Reunanen *et al.*	1995	None	[48]
	van Asperen *et al.*	1995	None	[49]
	Corti *et al.*	1997	Inverse	[27]
	Marniemi *et al.*	1998	None	[50]
	Manfroi *et al.*	1999	None	[51]
	Sempos *et al.*	2000	None	[52]
	Ascherio *et al.*	2001	None	[53]
	Knuiman *et al.*	2003	None	[54]
	van der A *et al.*	2006	Inverse	[30]
	Friedrich *et al.*	2009	None	[55]
	Menke *et al.*	2012	None	[57]
	Kim *et al.*	2012	Inverse	[32]
Clinical Trial	Zacharski *et al.*	2007	None	[33]
Meta-analysis	Danesh *et al.*	1999	None	[58]

We have analyzed 55 studies, of which 27 supported the iron hypothesis, 20 found no evidence to support it and eight were contrary to the iron hypothesis. We have found a clear support of the iron hypothesis in cross-sectional studies (nine of the 10 studies analyzed were in favor of it). Five of 15 case-control studies and nine of 24 cohort studies supported the iron hypothesis. Furthermore, three case-control studies and five cohort studies found an association opposite to the iron hypothesis. Concerning clinical-trials, four of the five studies revised supported the iron hypothesis. The meta-analysis showed no evidence in favor of the iron hypothesis in any of the studied iron biomarkers. These results suggest that there is not a high level of evidence which support the hypothesis that the iron may be associated with CVD. This disparity in results may be due to different iron biomarkers used in the studies. Despite the large number of studies published to date, the role of iron in CVD still generates a fair amount of debate. Future studies are needed to clarify the true effect of iron on CVD.
